# Importance of human demographic history knowledge in genetic studies involving multi-ethnic cohorts

**DOI:** 10.12688/wellcomeopenres.14692.3

**Published:** 2018-10-31

**Authors:** Benard W. Kulohoma

**Affiliations:** 1 Centre for Biotechnology and Bioinformatics, University of Nairobi, Nairobi, Kenya

**Keywords:** Africa, GWAS, Population substructure, H3Africa

## Abstract

Paucity of data from African populations due to under-representation in human genetic studies has impeded detailed understanding of the heritable human genome variation. This is despite the fact that Africa has sizeable genetic, cultural and linguistic diversity. There are renewed efforts to understand health problems relevant to African populations using more comprehensive datasets, and by improving expertise in health-related genomics among African scientists. We emphasise that careful consideration of the sampled populations from national and within-continental cohorts in large multi-ethnic genetic research efforts is required to maximise the prospects of identifying and fine-mapping novel risk variants in indigenous populations. We caution that human demographic history should be taken into consideration in such prospective genetic-association studies.

## Introduction

The 1000 Genomes Project (1000GP) is an invaluable resource that has improved understanding of global human genetic variation and its contribution to disease biology across multiple populations of distinct ethnicity ^1^. This catalogue of over 88 million high-quality variants from 26 populations has enhanced power to screen for common and rare variants that depict geographic and demographic differentiation ^2^. This represents 80% (approximately 80 million) of all variants contributed or validated in the public dbSNP catalogue, with recent major enhancements for genetic variation within several South Asian and African populations (24% and 28% of novel variants respectively) ^2^. Most of the low-frequency (< 0.5%) variants likely to be of functional significance are disproportionately present in individuals with substantial African ancestry, indicating bottlenecks in non-African populations ^2, 3^. The *“Luhya in Webuye, Kenya”* (LWK) population has the most accentuated number of these rare variants.

Paucity of data from African populations has restricted understanding of the heritable human genome variation. Although under-represented in human genetic studies, Africa has sizeable genetic, cultural and linguistic diversity (> 2000 distinct ethno-linguistic groups) ^4^. African populations are more genetically diverse, with considerable population substructure, and lower linkage disequilibrium (LD) compared to non-African populations ^4, 5^. Inclusion of more African populations will improve understanding of genetic variation attributed to complex population history, variations in climate, lifestyles, exposure to infectious diseases, and diets ^4, 6^. Diverse multi-ethnic imputation panels will undoubtedly improve fine-mapping of complex traits and provide detailed insights on disease susceptibility, drug responses, and improve therapeutic treatments. One such integrated panel, consisting of the phase 1 1000GP and African Genome Variation Project (AGVP) whole genome sequence panels, has shown marked improvement in detecting association signals in specific African populations poorly represented in the 1000GP ^7^. AGVP also present a new genotype array design that captures genetic variation in African populations.

**Figure 1.**
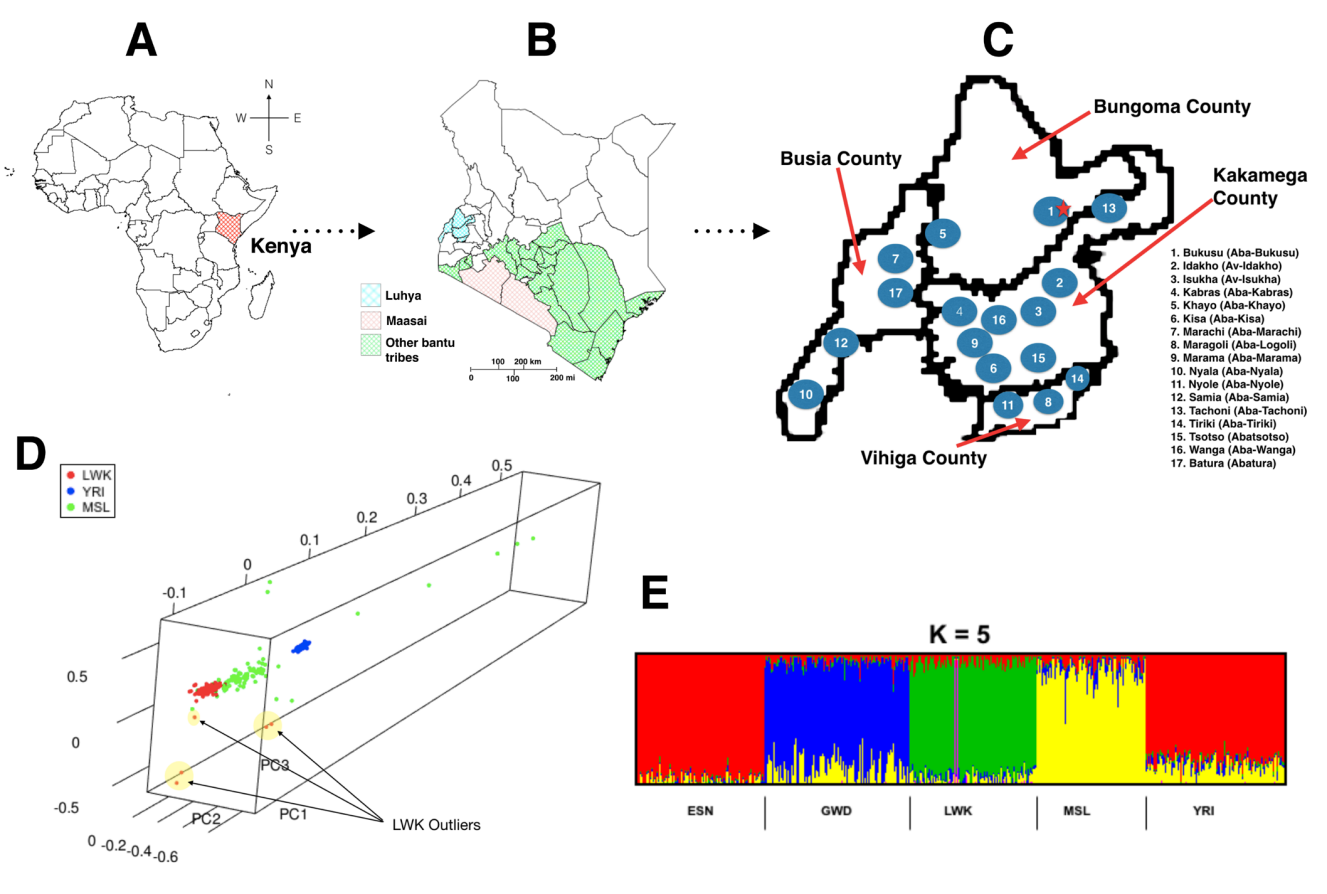
Population structure of the *Luhya in Webuye*, Kenya. ( **A**) Map of Africa showing the location of Kenya. ( **B**) Map of Kenya showing the location of Western Kenya Counties. ( **C**) The four Counties in Western Kenya inhabited by the “ *Luhya people*”. The 17 tribes of the Luhya, and the locations they hail from in Western Kenya are shown with numbers 1 through 17. ( **D**) The distribution of individuals from the LWK, YRI and MSL populations along the first three principal components. ( **E**) Ancestry for K value 5 for the Luhya (LWK) from Webuye, Kenya (n=99); Yoruba (YRI) from Ibadan, Nigeria (n=108); Esan (ESN) from Nigeria (n=99); Mandika (GWD) from The Gambia (n=113); and the Mende (MSL) from Sierra Leone (n=85)) examining the same 193,634 variants. The plot of ancestry fractions shows population sub-structure in the LWK population, when compared to five other populations from the 1000 Genomes Project (1000GP).

The Human Heredity and Health in Africa (H3Africa) initiative is aimed at understanding health problems relevant to African populations, and tilting the scales of data deficit and lacking expertise in health-related genomics among African scientists ^8, 9^. The H3Africa consortium consists of over 500 members, from more than 30 of the 55 African countries. H3Africa projects are focused on establishing genetic and environmental determinants associated with infectious (human African trypanosomiasis, tuberculosis, HIV, and other respiratory tract infections) and non-communicable diseases (kidney disease, diabetes, and cardiovascular diseases) ^10^. H3Africa is driven by African investigators, and is anticipated to close the gaps of ‘missing’ heritability by increasing the number of causal variants identified within genes, from a dataset of over 70,000 individuals collected using standardized protocols ^8, 10^. This presents a unique opportunity for the investigators to not only develop and direct their independent research agendas, but also enrich the datasets using their extensive knowledge of the continent’s history. However, careful consideration of the sampled populations in similar projects is required to maximise the prospects of identifying and fine-mapping novel risk variants in indigenous populations. In order to translate genomic research findings to useful resources for clinicians and drug development, substantial knowledge about reference populations that are relevant to the individuals being treated alongside the actionable variants is required ^10^. This is in addition to harmonised and well curated phenotype data that will allow easy integration and direct comparison of data outputs across different cohorts and phenotypes. Attentiveness to the considerable genetic substructure in African populations may reveal uncaptured variation and distinct ancestry ^11^. This extensive genetic diversity would benefit from strategies that explore genomics datasets that put local populations in context to provide more detail from disease mapping efforts in Africa. An example is the LWK in the 1000GP who do not represent all the *“Luhya people”*, a Bantu-speaking Niger-Congo population with a complex population history composed of 17 tribes, each with a distinct dialect ( Figure 1A – C) ^12, 13^. We examined for possible substructure in LWK, from 1000GP, to establish its implication on association studies.

## Methods

We used principal component analysis (PCA) to examine relationships within the Luhya (LWK) from Webuye, Kenya, population (n=99) using 193,634 variants from the 1000GP phase 3 ^2^. We compared LWK to African populations from the 1000GP phase 3 (Yoruba (YRI) from Ibadan, Nigeria (n=108); Esan (ESN) from Nigeria (n=99); Mandika (GWD) from The Gambia (n=113); and the Mende (MSL) from Sierra Leone (n=85)) examining the same 193,634 variants, since these populations also speak the Niger-Kordofanian languages, and share recent genetic ancestry ^12, 14^. The 1000GP call set was already filtered down using VCFtools (v 0.1.12b) and PLINK (v1.90b6.2), and only contained biallelic, non-singleton SNV sites that are a minimum of 2KB apart from each other and a minor allele frequency > 0.05 ^2, 15, 16^. We considered just the first three principal components (PCs) computed to resolve the population substructure. We then used ADMIXTURE (v1.3) to estimate ancestry for K values from 2 through 20 ^17^. Distruct plots of the output ancestry fractions were generated using Genesis (v 0.2.6b) ^18^.

## Results and discussion

Our PCA analyses reveal that all individuals in the LWK population cluster closely except five individuals along PC2 (n=2) and PC3 (n=3), possibly suggesting that the outliers are individuals from different Luhya tribes ( Figure 1D, & Supplementary Figure 1 & Supplementary Figure 2). We suggest that whereas the first principal component, PC1, distinguished individuals primarily on genetic ancestry, PC2 and PC3 may reflect genetic diversity associated with differences in the geographic distribution and linguistic differences of the individuals. We propose that although a huge proportion of individuals in the LWK population are actually from Webuye, which predominantly inhabited by the Bukusu tribe, the outliers hail from various other settlements associated with other Luhya tribes ( Figure 1C). Unsupervised ADMIXTURE analysis suggests minimal substructure ( Figure 1E, & Supplementary Figure 3).

In sub-Saharan African (SSA), there are nearly 500 closely related but distinct languages distributed over a total area of approximately 500 000 km ^2^ ^14^. These languages are spoken by approximately one quarter of the SSA population (~200 million people) ^14, 19^. The Bantu languages fall into this category, and consist of separate groups that constitute part of the Niger-Congo language phylum ^20^. The spread of Bantu-speaking populations in SSA is primarily due to historical migration of populations, approximately 3000–5000 years ago, and not solely due to diffusion of language ^14^. This demographic history is associated with admixture and changes in population structure, resulting in complex patterns of genetic variation in present day populations ^21, 22^. An example is the identification of haplotypes among Nilo-Saharan language speakers of the Luo community that neighbours the Luhya of Western Kenya, which were previously thought to be private in Bantu populations, that are now associated with interactions between these distinct populations during the migration of the Bantu farmer populations ^22^. Previous studies on Bantu expansion and migration suggest populations first moved south from their homeland, near the Nigeria-Cameroon border, through the rainforest and split into two groups: one branched south and west; while another moved east towards the Great Lakes ^14, 23^. The East Bantu languages, which also include the Luhya language, are distributed in East and Southern Africa ^23^. In Kenya, these Eastern Bantu speaking populations are further categorised into two based on their migratory routes to present day Kenya: the Eastern Kenya Bantus (Kamba, Kikuyu, Meru, Embu, Taita, Giriama, Kombe, Chonyi, Digo, Rabai, Jibana, Pokomo, Duruma, Kauma and Ribe) and Western Kenya Bantus (Kisii, Luhya, Kuria, Suba and Khene) ^24, 25^.

Multi-disciplinary approaches that enlist the knowledge of anthropologists, linguists, geneticists, and historians would significantly improve understanding on human history and migration of populations, genetics of complex traits and adaptive variations to modern environments, and language and cultural changes ^26– 28^. Previous studies on intricate languages in China, and Australia suggest consistency of genetic and linguistic evolution, with striking evidence of compatible phylogenetic signal and phonological evolution ^29, 30^. In SSA such studies are hindered by paucity of data with only a limited number of reasonably close populations available, impeding more detailed analysis ^18, 31^. A recent study highlights population differentiation between two South Eastern Bantu groups in South Africa, which were assumed to be genetically homogenous, further emphasising the importance of having a clear perspective of population structure in disease-association studies ^18^. This result was arrived at by understanding ethnolinguistic divisions within the present-day population, and purposely recruiting from rural areas or regions with little ethnolinguistic diversity ^18^.

The multi-ethnic genetic-association studies, like those in the H3Africa initiative, now offer a unique opportunity to resolve this challenge using multiple large scale GWAS analyses of important genetic traits from diverse populations across Africa. GWAS studies largely rely on self-reported data on ethnic background. Genetic information is then used to confirm ancestral backgrounds and exclude outliers. However, this may lead to insufficient representation of some populations, and disease-association studies of low prevalence or late onset conditions, such as Alzheimer’s disease, would be underpowered. Thus, in order to understand complex traits in say the entire “ *Luhya people*”, adequate sampling of underrepresented tribes would provide a high-resolution view of their ancestral history. Haphazard sampling would significantly reduce power to detect signal due to population substructure, even within this single community. We speculate that this was largely circumvented at recruitment when sampling LWK in the 1000GP by asking the participants whether all four of their grandparents were of the Bukusu tribe. Whereas projects covering relatively small geographical areas are able to overcome such challenges, national and within-continental cohorts in large multi-ethnic genetic research efforts must have well thought out documented protocols that carefully consider human demographic history.

## Data availability

The LWK, ESN, GWD, MSL, and YRI datasets were obtained from the European Bioinformatics Institute 1000 Genomes Project website http://ftp.1000genomes.ebi.ac.uk/vol1/ftp/release/20130502/supporting/admixture_files/

## Supplementary material

**Supplementary Figure 1**

The distribution of individuals from the LWK, YRI, MSL, ESN and GWD populations along the first three principal components.

**Supplementary Figure 2**

The distribution of individuals from only the LWK, and YRI populations along the first three principal components.

**Supplementary Figure 3.**

Ancestry for K values 2 through 7 for: (A) The Luhya (LWK) from Webuye, Kenya (n=99); Yoruba (YRI) from Ibadan, Nigeria (n=108); and the Mende (MSL). (B) The Luhya (LWK) from Webuye, Kenya (n=99); Yoruba (YRI) from Ibadan, Nigeria (n=108); Esan (ESN) from Nigeria (n=99); Mandika (GWD) from The Gambia (n=113); and the Mende (MSL) from Sierra Leone (n=85)). The same 193,634 variants were examined in all analyses. The plot of ancestry fractions shows population sub-structure in the LWK population, when compared to five other populations from the 1000GP.
