# Supplementary figures and images for: Importance of human demographic history knowledge in genetic studies involving multi-ethnic cohorts
Source: Wellcome Open Res. 2018 Oct 31;3:82. Originally published 2018 Jul 6. [Version 3] doi: 10.12688/wellcomeopenres.14692.3 (PMC6206618; doi:10.12688/wellcomeopenres.14692.3)

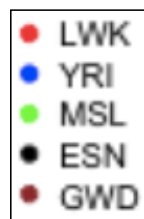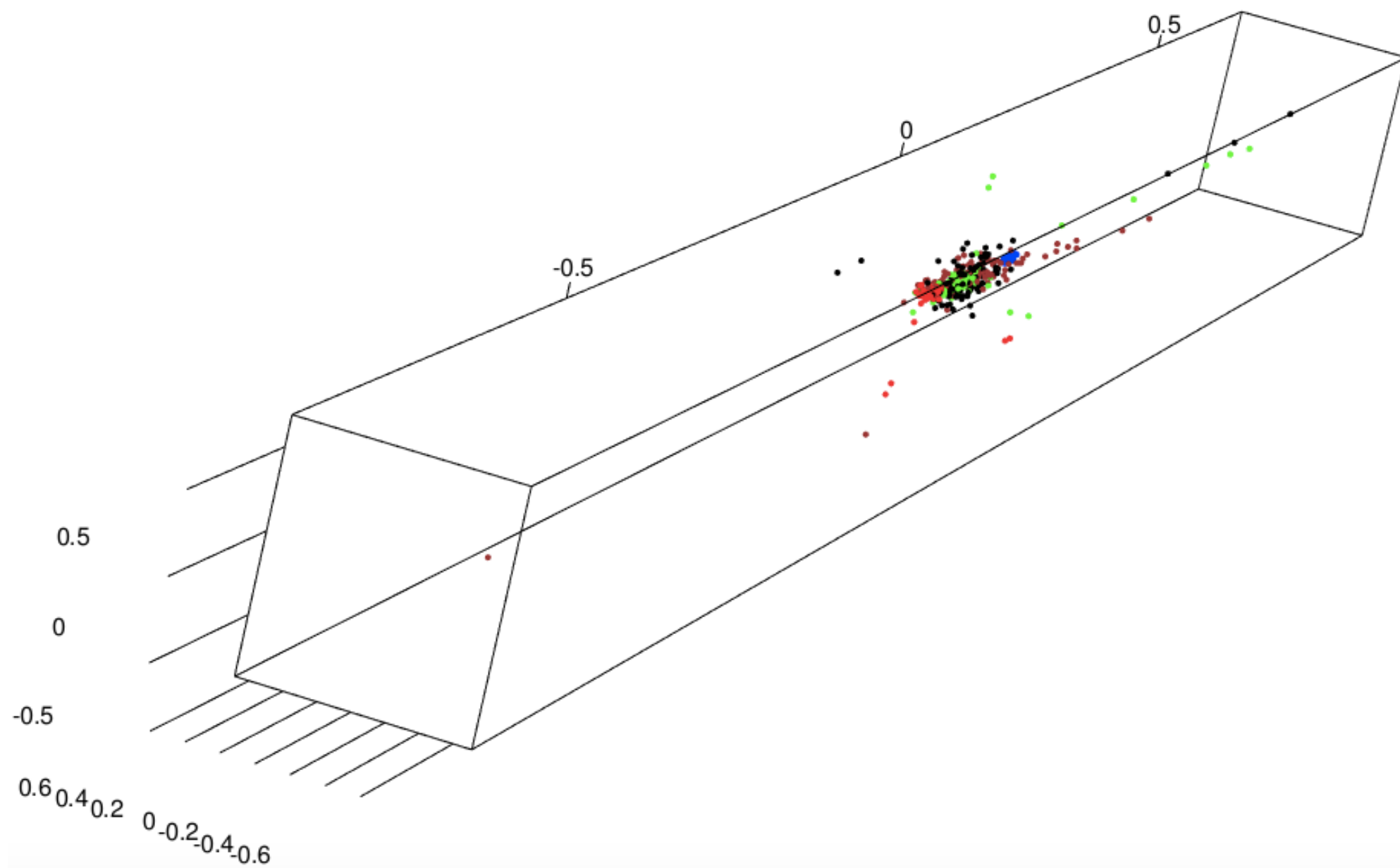

Supplement: Supplementary file 1 [file wellcomeopenres-3-16251-s0000.tgz › a20d6254-cd07-4ea6-87f0-c8e671f6a681_Supplementary_Figure_1.pdf]

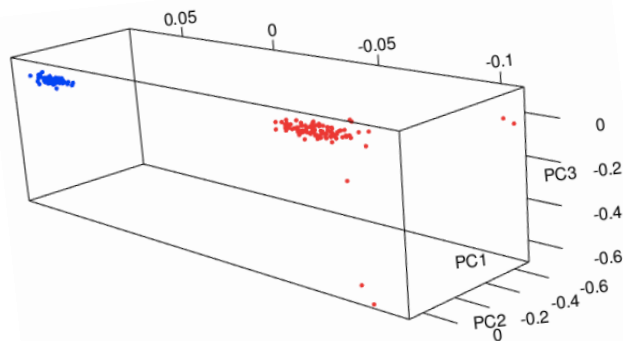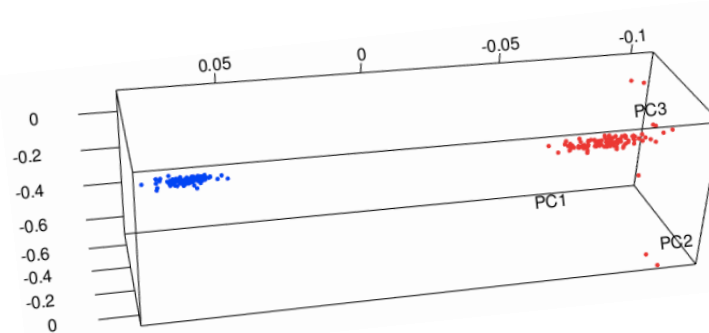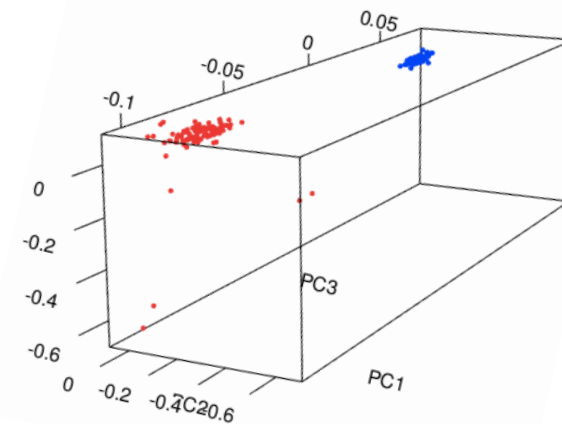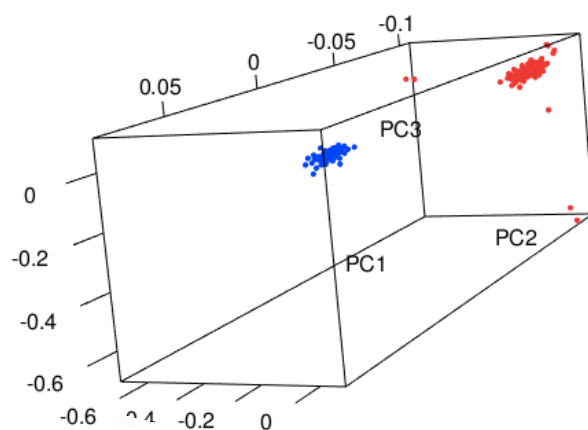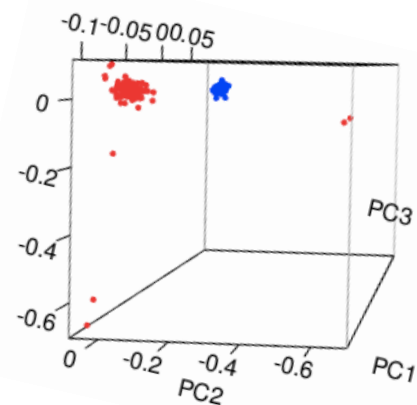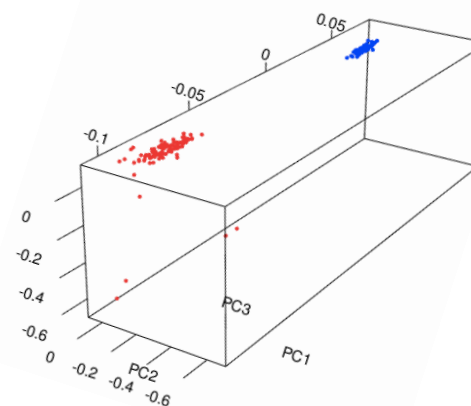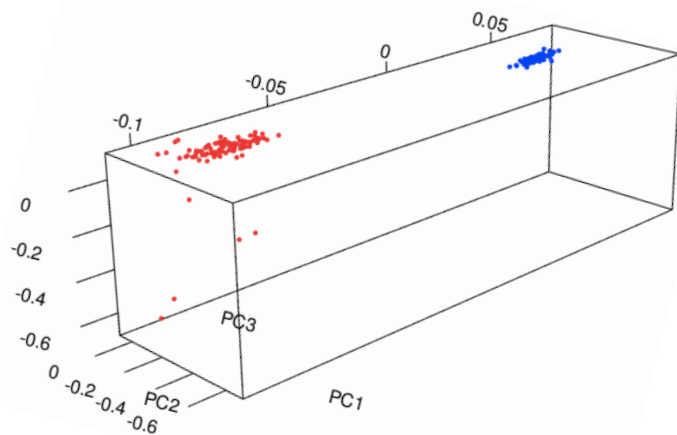

Supplement: Supplementary file 2 [file wellcomeopenres-3-16251-s0001.tgz › 8ba9461c-6d8d-4588-917b-fe97a7ad9e7f_Supplementary_Figure_2.pdf]

**A**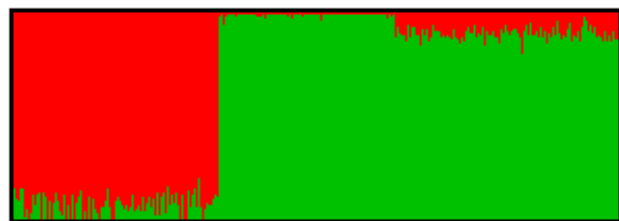**K = 2**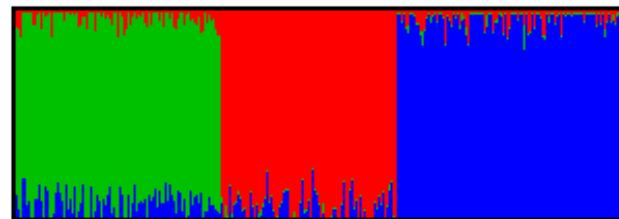**K = 3**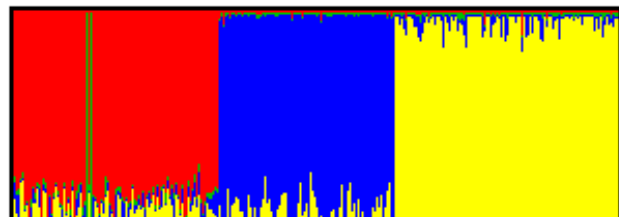**K = 4**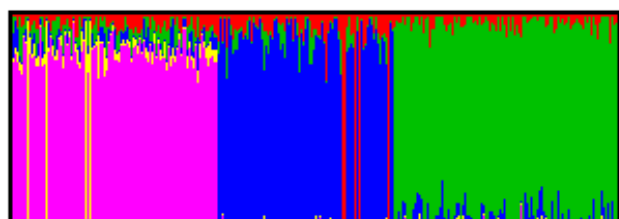**K = 5**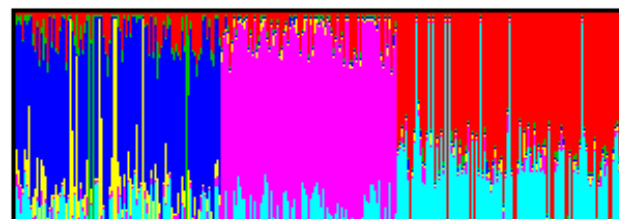**K = 6**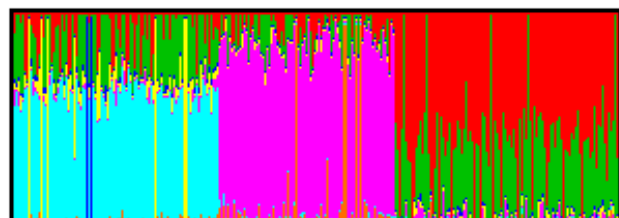**K = 7**

LWK

MSL

YRI

**B**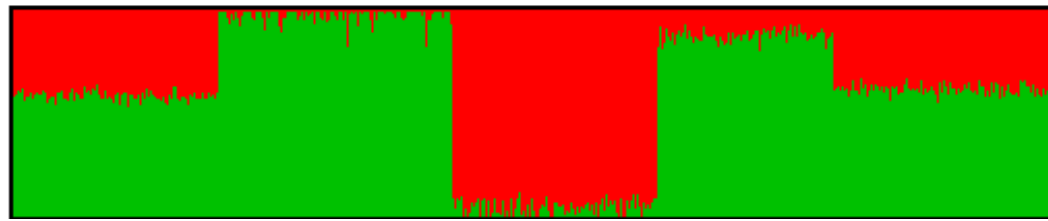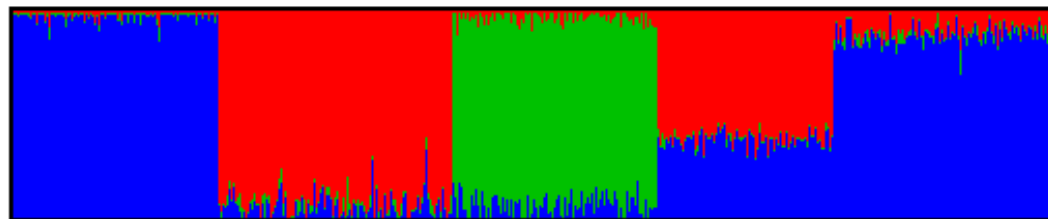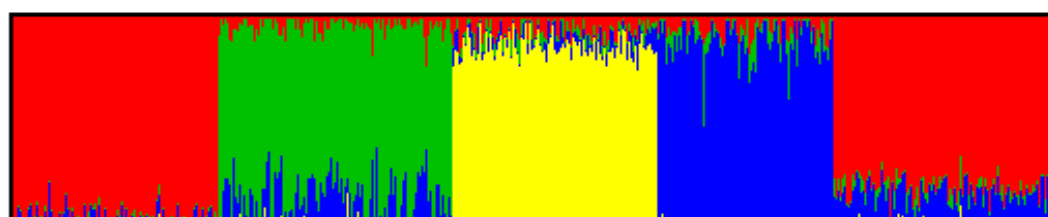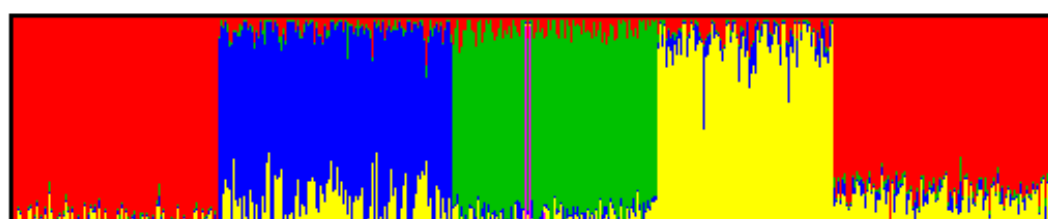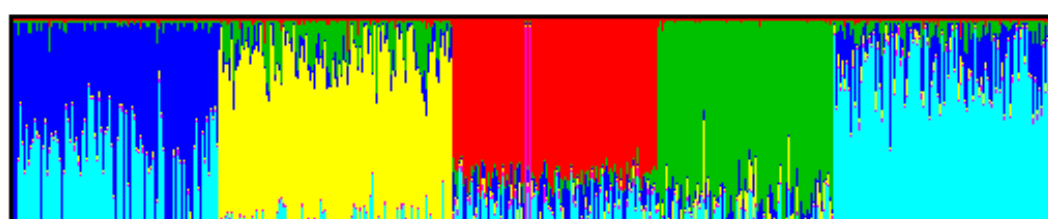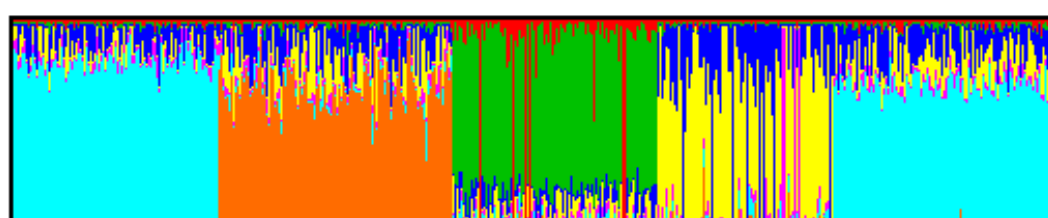

ESN

GWD

LWK

MSL

YRI

Supplement: Supplementary file 3 [file wellcomeopenres-3-16251-s0002.tgz › 650d4ece-192f-4fe2-b478-9cfd256f8eb1_Supplementary_Figure_3.pdf]
