# Supplementary material for: Importance of human demographic history knowledge in genetic studies involving multi-ethnic cohorts
Source: Wellcome Open Res. 2018 Oct 31;3:82. Originally published 2018 Jul 6. [Version 3] doi: 10.12688/wellcomeopenres.14692.3 (PMC6206618; doi:10.12688/wellcomeopenres.14692.3)
Supplement: Peer review history

# Peer review: Referee response for version 3

**DOI:** 10.21956/wellcomeopenres.16251.r34141

**Published:** 2018-11-01

**Authors:** Michèle Ramsay

**Affiliations:** 1 Sydney Brenner Institute for Molecular Bioscience, Division of Human Genetics, Faculty of Health Sciences, University of the Witwatersrand, Johannesburg, South Africa

I have read this submission. I believe that I have an appropriate level of expertise to confirm that it is of an acceptable scientific standard.

# Peer review: Referee response for version 2

**DOI:** 10.21956/wellcomeopenres.16146.r33899

**Published:** 2018-10-24

**Authors:** Nicola J. Mulder

**Affiliations:** 1 Computational Biology Group, Department of Integrative Biomedical Sciences, Institute of Infectious Disease and Molecular Medicine, University of Cape Town, Cape Town, South Africa

I am still concerned that the content is a bit thin for a research paper, but as a short research note perhaps it is sufficient. I suggest the abstract is updated to not just mention cautions but to add a couple of sentences about what the paper aimed to do and the key findings before the cautionary comments.

I have read this submission. I believe that I have an appropriate level of expertise to confirm that it is of an acceptable scientific standard.

# Peer review: Referee response for version 2

**DOI:** 10.21956/wellcomeopenres.16146.r33898

**Published:** 2018-10-02

**Authors:** Michèle Ramsay

**Affiliations:** 1 Sydney Brenner Institute for Molecular Bioscience, Division of Human Genetics, Faculty of Health Sciences, University of the Witwatersrand, Johannesburg, South Africa

The new title is appropriate and the paper is much improved.

 This is a single author paper so the attribution to “we” should be “I”. Please check throughout or revise sentences.

 Introduction:

 First paragraph: It is unclear what “80% (80 million) of all variants” in dbSNP is based on. The previous sentence refers to 88 million variants. Reference 2 was published in 2015 and therefore is no longer current as dbSNP has increased considerably.

 Some minor editing would be beneficial

 Introduction second paragraph: Second last sentence requires revision.

 Third paragraph: line 11 from the bottom of the page – “population” should be “populations” may reveal…

 The multi-ethnic genetic-association studies, like those in the

 H3Africa initiative, now offers (should be “offer”) a unique opportunity…..

 Data availability: Also include the other populations from 1000GP that were used in the study.

I have read this submission. I believe that I have an appropriate level of expertise to confirm that it is of an acceptable scientific standard.

# Peer review: Referee response for version 1

**DOI:** 10.21956/wellcomeopenres.15998.r33497

**Published:** 2018-08-06

**Authors:** Nicola J. Mulder

**Affiliations:** 1 Computational Biology Group, Department of Integrative Biomedical Sciences, Institute of Infectious Disease and Molecular Medicine, University of Cape Town, Cape Town, South Africa

This paper uses the LWK population from the 1000 Genomes dataset to demonstrate the need for considering demographic data when sampling. The paper is very thin on results for a research paper as it only really includes a PCA on a small sample set. In order for it to be a reasonable research paper it would need to include other populations and data to verify the findings. It is also not clear to me whether there is support for the ethnic origin of the samples, is this information available?

 Additional comments:

- The title refers to H3Africa but the paper is about the 1000 Genomes data so I recommend that the title changes, the results are relevant to all studies, not just H3Africa.
- In the abstract and later there is mention of "titling" was this supposed to "tilting"?
- There are too many figures to demonstrate a simple point.

Though the message of the paper is important, I think it needs more work. The PCAs and the paper in general need to include more populations to verify the point.

I have read this submission. I believe that I have an appropriate level of expertise to confirm that it is of an acceptable scientific standard, however I have significant reservations, as outlined above.

# Response

**Published:** 2018-09-10

**Authors:** Benard Kulohoma

We have made the following changes to this research note paper:

 We have revised the analysis to include more populations in the analysis to verify our findings. We now also include anthropology and population genetics references on the history of the Bantu migration, and the Luhya population of Western Kenya. We have made revisions to the analysis, and now include other populations that share recent genetic ancestry from the 1000GP that belong to the Niger-Kordofanian Bantoid language group for comparison.

 We have made revisions to the title to encompass all studies, not just H3Africa.

 We have amended the word "titling" to "tilting". We have also revised the manuscript and reduced the number of figures.

 We have made revisions to the analysis, and include other populations that share recent genetic ancestry from the 1000GP that belong to the Niger-Kordofanian language group for comparison.

# Peer review: Referee response for version 1

**DOI:** 10.21956/wellcomeopenres.15998.r33495

**Published:** 2018-08-03

**Authors:** Michèle Ramsay

**Affiliations:** 1 Sydney Brenner Institute for Molecular Bioscience, Division of Human Genetics, Faculty of Health Sciences, University of the Witwatersrand, Johannesburg, South Africa

This study uses genotype data from the 1000 Genomes Project to examine population structure among the 99 individuals of the Luhya people sampled from Webuye in Kenya. It emphasizes the need for careful participant selection in disease-related genome-wide studies in African populations. Principal component analysis (PCA) and ADMIXTURE are used. I fully agree that we need to consider demographic histories when analyzing genomic data, but found the title misleading in two ways: Why focus on H3Africa as the target audience and not more generally research among Africans (and other populations) and, secondly, how does selection of this group of Luhya people demonstrate that the sampling was less than optimal. The only suggestion in the paper, related to the title, is to request data on the ethnolinguistic origins of the grandparents during the participant selection phase – I was expecting more insight into how to include knowledge of demographic history into a sampling framework (e.g. collaborate or consult with anthropologists, linguists and historians; select participants from rural areas that have less ethnolinguistic diversity and admixture; request ethnolinguistic identification of grandparent; etc.). Sampling frameworks will of course be influenced by the aims and objectives of the research.    

 The study focused on a single population and no additional or neighboring populations were included in the PCA and ADMIXTURE analyses.  It is therefore unclear what the three ancestral components (K=3) are likely to be and what the origins of the 5 outliers may be. The sampling appears to have been good in identifying a closely related group of individuals, which is not unexpected as they were from a single ethnolinguistic origin and geographic location. It would be very interesting to sample all 17 Luhya tribes to determine whether they are genetically distinguishable on PCA, but I appreciate that these data are not available. An understanding of their demographic history could provide some clues as to what one may expect. Perhaps refer to the recent paper on South African genomes by Choudhury et al. ^1^.

 Currently there are good analysis programs that either rely on adjusting for ancestral component diversity or using meta-analysis when participants are from significantly different populations (e.g. GEMMA, METAL, BOLT-LMM and others). Since there is a vast amount of data on every participant in GWAS studies, PCA can effectively be used to identify outliers and to exclude them, if appropriate, or adjust for population sub-structure.

 The paper would be strengthened by providing other examples and illustrating how some studies/populations have been successful at using demographic history in their sampling strategy whereas others fall short, as they have ignored this. The figures are numerous and do not all add additional value, no need to show structure plots at high K values. The structure plots would be enhanced by using a tool such as Genesis ( www.bioinf.wits.ac .za/software/genesis) to ensure that the individuals are shown in the same order and that the same color is used throughout to represent the same ancestral component for the different K values.

 There are several sentences that could be rewritten to improve meaning and direct repetition of whole sentences should be avoided between the abstract and paper content. Presumably the author meant “tilting the scales” and not “titling the scales”?

I have read this submission. I believe that I have an appropriate level of expertise to confirm that it is of an acceptable scientific standard, however I have significant reservations, as outlined above.

## References

[1] ChoudhuryARamsayMHazelhurstSAronSBardienSBothaGChimusaERChristoffelsAGamieldienJSefid-DashtiMJJoubertFMeintjesAMulderNRamesarRReesJScholtzKSenguptaDSoodyallHVenterPWarnichLPepperMS: Whole-genome sequencing for an enhanced understanding of genetic variation among South Africans. *Nat Commun*.2017;8(1) : 10.1038/s41467-017-00663-9 2062 10.1038/s41467-017-00663-9 29233967PMC5727231

# Response

**Published:** 2018-09-10

**Authors:** Benard Kulohoma

We have revised the title to make it more general to encompass all studies, not just H3Africa. We have made revisions to illustrate that these considerations should be made to all multi-ethnic GWAS studies.

 We have amended the manuscript and discuss the importance of multi-disciplinary approaches that enlist the knowledge of anthropologists, linguists, geneticists, and historians to improve understanding on human history and migration of populations, genetics of complex traits and adaptive variations to modern environments, and language and cultural changes.     

 We have made revisions to the analysis, and include other populations that share recent genetic ancestry from the 1000GP that belong to the Niger-Kordofanian language group for comparison.

 We agree that current analysis program that implement standard linear regression can use principal components from PCA analysis to avoid errors due to population stratification, as well as speed-up computation. Essentially, our manuscript is aimed at highlighting population stratification; and the importance of multi-disciplinary approaches when sampling underrepresented populations of interest to put local populations in the right context and provide more detailed and accurate information for disease mapping efforts in Africa. This will provide a more granular understanding on the genetic traits associated with these populations. Although GWAS studies largely rely on self-reported data on ethnic background, which is the verified using genetic information to confirm ancestral backgrounds and exclude outliers. The exclusion of individuals may lead to insufficient representation of some populations, and disease-association studies of low prevalence or late onset conditions, such as Alzheimer’s disease, would be underpowered. Taking population history into account during study design my help to alleviate these challenges.

 We have revised the manuscript to provide examples of how multi-disciplinary approaches could improve research findings.

 We have revised the manuscript to scale down the number of figures. We have also redrawn the structure plot.

 We have made revisions to the manuscript to remove repetitions and improve meaning. We have amended the word "titling" to "tilting".
